# Variation in measuring and ophthalmic factors correlating with central corneal thickness in the Northern Finland birth cohort eye study

**DOI:** 10.1111/aos.70163

**Published:** 2026-05-13

**Authors:** Tuomas Qvist, Oona Ahokas, Johanna Liinamaa, Ville Saarela

**Affiliations:** ^1^ Department of Ophthalmology, Medical Research Center, Oulu University Hospital and Research Group of Clinical Medicine University of Oulu Oulu Finland; ^2^ Department of Ophthalmology, Medical Research Center, Oulu University Hospital, and Department of Mathematical Sciences University of Oulu Oulu Finland

**Keywords:** central corneal thickness, cohort, intraocular pressure, optical coherence tomography, randomized, ultrasound pachymetry

## Abstract

**Purpose:**

To assess the correlation between optical coherence tomography (OCT) and ultrasound pachymetry (USP) as methods for analysing central corneal thickness (CCT) and to evaluate ophthalmic factors affecting CCT.

**Methods:**

The eyes of 3070 subjects of the Northern Finland Birth Cohort (NFBC) were examined at the age of 45–49 years. CCT was measured using OCT and USP. The optic nerve head (ONH) and retinal nerve fibre layer (RNFL) parameters were assessed with OCT and Heidelberg Retina Tomograph (HRT). Visual acuity and refraction were evaluated with an autorefractometer and intraocular pressure (IOP) with rebound tonometry (iCare) and Goldmann applanation tonometry (GAT). The evaluation of glaucomatous damage was based on the ONH, RNFL and visual fields.

**Results:**

The mean CCT was 540.0 ± 34.8 μm with OCT and 540.7 ± 39.6 μm with USP. There was a strong correlation (*r* = 0.927, *p* < 0.001) between the devices, and the inter‐method agreement increased with better visual acuity (*r* = 0.086, *p* = 0.003). The CCT measured with OCT correlated with IOP (*r* = 0.400 (iCare), *r* = 0.314 (GAT), *p* < 0.001), rim area (*r* = 0.048, *p* = 0.011), cup volume (*r* = −0.046, *p* = 0.014) and RNFL (*r* = 0.041, *p* = 0.027). We found no correlation between CCT and glaucoma.

**Conclusion:**

The results indicate that OCT and USP may be used interchangeably when measuring CCT. As expected, CCT had a substantial correlation with IOP, but was also positively associated with ocular neural tissue (rim and RNFL) and negatively with the optic cup.

## 1 INTRODUCTION

The measurement of central corneal thickness (CCT) is a routine procedure in an ophthalmic examination. The gold standard for measuring CCT is currently ultrasound pachymetry (USP), but optical coherence tomography (OCT) and other methods are also used. The precise measurement of corneal thickness is clinically significant, for example, when planning refractive procedures (Wirbelauer et al., 2000), in the evaluation and treatment of keratoconus (Santodomingo‐Rubido et al., 2022) and in the assessment of intraocular pressure (IOP) (European Glaucoma Society, 2021).

A study by Doğan & Ertan found significant inter‐method differences in measuring CCT, including a relatively high (17.4 μm) mean difference between USP and OCT (Doğan & Ertan, 2019). However, other recent studies have shown a good correlation between OCT and USP measurements, with mean differences below 1 μm (Gokcinar et al., 2019; Ucak et al., 2021). CCT is associated with IOP in both glaucoma patients (Doughty & Zaman, 2000) and healthy subjects (Soleimanizad et al., 2017). Lower CCT has been found to be a predictive factor for the development and progression of primary open‐angle glaucoma (POAG) in patients with ocular hypertension (OHT) (Gordon et al., 2002). It has been shown that patients with POAG have a 2.7 times higher probability of a CCT below 515 μm (Fernandez‐Bahamonde et al., 2011). However, a recent simulation of the data suggests that the correlation between CCT and glaucoma may be due to collider bias and CCT cannot be used to identify people at risk of glaucoma in the general population (Khawaja & Jansonius, 2022). Also, a lack of association between CCT and glaucoma has been described (Bikbov et al., 2023). CCT has shown variable correlation with a number of optic nerve head (ONH) and retinal nerve fibre layer (RNFL) parameters in different studies. In general, CCT has demonstrated significant correlation with larger rim area and RNFL thickness (Gunvant et al., 2008; Viswanathan et al., 2013), but also insignificant (Wu et al., 2011) and even significant opposite results (Cankaya et al., 2008) have been published.

There are contradictory results in the literature on how uniform the methods for measuring CCT are (Doğan & Ertan, 2019; Ucak et al., 2021). Furthermore, there is a lack of knowledge on the correlation between CCT and ophthalmic parameters in the normal population. Additionally, there is very limited information on whether these parameters have an influence on the inter‐method agreement of measuring CCT. These methods are based on different physical properties, and pachymetry requires contact with the cornea. We hypothesized that changes in ophthalmic parameters may reflect differently on optical measurements compared with ultrasound.

The present study evaluates CCT in a randomized middle‐aged birth cohort from Northern Finland. The purpose of this study is to assess the inter‐method agreement of USP and OCT and to explore the relationships of both CCT and the inter‐method agreement with ophthalmic parameters.

## 2 METHODS

### 2.1 Study population

The Northern Finland Birth Cohort (NFBC) Eye Study was conducted as a randomized prospective cohort study (Saarela et al., 2013). The original NFBC population consists of 12 231 subjects (covering 96% of the deliveries) from the provinces of Oulu and Lapland in Northern Finland. Subjects of the NFBC are individuals who had an expected date of birth in 1966. The follow‐up has been ongoing for the 12 058 liveborn subjects since 1966 (Nordström et al., 2022). In 2011, 10 331 of the subjects (84% of the original cohort) were alive and had an address in Finland.

Half of the cohort population (5155 subjects) was randomly selected to the examination group for the NFBC Eye Study. The other half served as controls. Randomization was performed for gender, area of residence and month of birth using the Resampling Stats software (Resampling Stats Inc., Arlington, VA, USA). A total of 3070 (60%) subjects aged 45–49 years in the examination group attended the eye examination between 2012 and 2015. The right eye for each subject was chosen for the present analysis. Approval for the study has been granted by the Ethical Committee of the Northern Ostrobothnia Hospital District. Written informed consent was acquired from all participating subjects.

### 2.2 CCT and IOP measurements

CCT was measured with an ultrasonic Tomey pachymeter SP‐3000 (Tomey Co., Nagoya, Japan) and the Anterior Segment HD Images protocol of Cirrus HD‐OCT 4000 (software version 6.0.0; Carl Zeiss Meditec AG, Oberkochen, Germany). Pachymetry data is available for the subjects examined in Oulu University Hospital. Anterior segment OCT was available for all the subjects, also those examined in remote locations. IOP was assessed using rebound tonometer iCare TA01i (Icare Ltd., Vantaa, Finland) and the Goldmann applanation tonometer (GAT).

### 2.3 Refraction and visual acuity

Objective refraction, including mean spherical equivalent (MSE), was measured using the Nidek AR‐360A autorefractometer (Nidek Co, Ltd., Gamagori, Japan). The ETDRS chart was used to measure best corrected visual acuity.

### 2.4 ONH and RNFL imaging

Canon CF‐60DSi Digital Mydriatic Fundus Camera with attached Canon EOS‐1Ds MK III SLR Digital Camera (Canon Inc., Tokyo Japan) was used to obtain colour and grayscale digital fundus images. The pupils were dilated for imaging. The images were processed with Adobe Photoshop CS (Adobe Systems Inc., San Jose, California, USA) and Neacapture software (Neagen Ltd., Oulu, Finland). We used the blue monochromatic interference filter when capturing the RNFL images with the camera specified above. A skilled photographer, trained for this task, captured and processed the RNFL images. Two general ophthalmologists, working independently, performed the screening evaluations of the RNFL and ONH images in a masked fashion.

Optic nerve head topography was measured using the Heidelberg Retina Tomograph (HRT3; Heidelberg Engineering, Heidelberg, Germany). The device captures a series of images at different depths to create a three‐dimensional topography image. Stereometric ONH parameters, such as disc area, rim area and cup volume, are derived from this image. RNFL and ONH measurements were also obtained with Cirrus HD‐OCT 4000 using the Optic Disc Cube 200 × 200 protocol.

### 2.5 Glaucoma diagnosis

The diagnosis of glaucoma was based on the evaluation of the ONH, RNFL and visual fields (VF). The ‘2 out of 3’ rule of the Finnish current care guideline (Tuulonen et al., 2003) was applied. Findings consistent with glaucomatous damage in at least two of the three separate tests (ONH, RNFL, VF) were required for the diagnosis of definite glaucoma. Glaucoma suspects showed glaucomatous findings in one of the three tests. Glaucoma was ruled out when the subject exhibited a normal ONH, RNFL and VF.

The size and shape of the ONH, cup shape and depth, neuroretinal rim and haemorrhages were evaluated from the stereo ONH photographs. For example, an ONH notch was considered glaucomatous. In contrast, large cupping alone with a normal and even‐width neuroretinal rim was not regarded as an abnormality when RNFL and VF were normal. The RNFL photographs were assessed for both local and diffuse RNFL defects, both of which were considered glaucomatous.

The evaluation of a glaucomatous field defect was based on the reduction in sensitivity in at least three clustered test points below the 5% level of which at least one was below the 1% level, glaucoma hemifield test (GHT) graded ‘outside normal limits’ or pattern standard deviation abnormal at *p* < 0.05 level.

Glaucomatous findings were further independently verified in a masked fashion by three glaucoma experts. The evaluation process has been previously described in detail (Karvonen et al., 2019).

### 2.6 Statistical analysis

The statistical analyses were performed with IBM spss Statistics for windows version 28.0 and the graphical line arts were produced with R language version 4.3.1. The right eye of the participants was chosen for the analyses. All eyes (normal, glaucoma suspect and glaucomatous) were included. The data of the present analysis was not randomized. The variables were visually explored before conducting any statistical tests, for example, distributions were examined using histograms and correlations were examined using scatter plot matrices. Mean and standard deviation (SD) are reported for normally distributed variables and median and interquartile range (IQR) for non‐normally distributed variables. Bland–Altman analysis was used to assess the correlation between OCT and USP measurements. Limits of agreement (LOA) were defined as mean ± 1.96 SD. Scatter plot images were generated to show the distribution of the CCT measurements with OCT and USP.

To assess the linear relationship between CCT values and ocular parameters, Pearson's correlation coefficients and related *p*‐values were calculated. Pearson's correlation coefficient was used to evaluate the correlations between CCT and different ophthalmic parameters: disc area, rim area, RNFL thickness and IOP. Spearman's rho was employed in relation to refraction and cup volume due to the non‐normal distribution of these variables. Linear regression between IOP (iCare) and CCT was calculated using CCT OCT as the independent variable. For other parameters that demonstrated significant correlation with CCT, multiple linear regression models were used to further quantify their predictive power using CCT as the dependent variable. The first model included only the ophthalmic parameters as independent variables. The second model was subsequently adjusted for gender and glaucoma status.

The difference and the absolute difference between the CCT measurements obtained by OCT and USP were calculated. To evaluate both systematic and random effects, the correlation analysis was performed between the ophthalmic parameters and both the CCT differences and the absolute differences. Welch's ANOVA test was used for the comparison of CCT mean values of glaucoma classes. Post‐hoc Games–Howell test was used for comparisons between glaucoma classes. A *p*‐value lower than 0.05 was considered statistically significant.

## 3 RESULTS

This study included 3069 participants, 1687 females (55%) and 1382 males. The mean age was 47.5 ± 0.9 years. The mean CCT was 540.0 ± 34.8 μm for OCT and 540.7 ± 39.6 μm for USP. The mean IOP was 14.9 mmHg with iCare. The number and distribution of the ophthalmic examinations are specified in Table 1.

**TABLE 1:** Statistics of the studied ophthalmic parameters.

Variable	*N*	Mean	SD
CCT (OCT) (μm)	2856	540.0	34.8
CCT (USP) (μm)	1412	540.7	39.6
Visual acuity/ETDRS*	3043	−0.08	−0.14 to 0.00
Disc area (mm^2^)			
OCT	2989	1.75	0.34
HRT	2980	2.18	0.47
Rim area (HRT) (mm^2^)	2980	1.73	0.34
Cup volume (HRT) (mm^3^)*	2980	0.06	0.02 to 0.14
RNFL (μm) (OCT)	3012	91.16	9.79
Refraction/mean spherical equivalent (D)*	3056	−0.38	−2.00 to 0.38
Intraocular pressure (mmHg)			
Goldmann Applanation Tonometry	1267	13.87	2.93
ICare	3057	14.94	3.48

Abbreviations: HRT, Heidelberg Retinal Tomograph; OCT, optical coherence tomography; RNFL, retinal nerve fibre layer.

* Median and interquartile range (IQR) for non‐normally distributed variable.

There was a strong correlation (*r* = 0.927, *p* < 0.001) between CCT values measured with OCT and USP. Scatter plot demonstrating the correlation is presented in Figure 1a and Bland–Altman analysis in Figure 1b.

**FIGURE 1:**
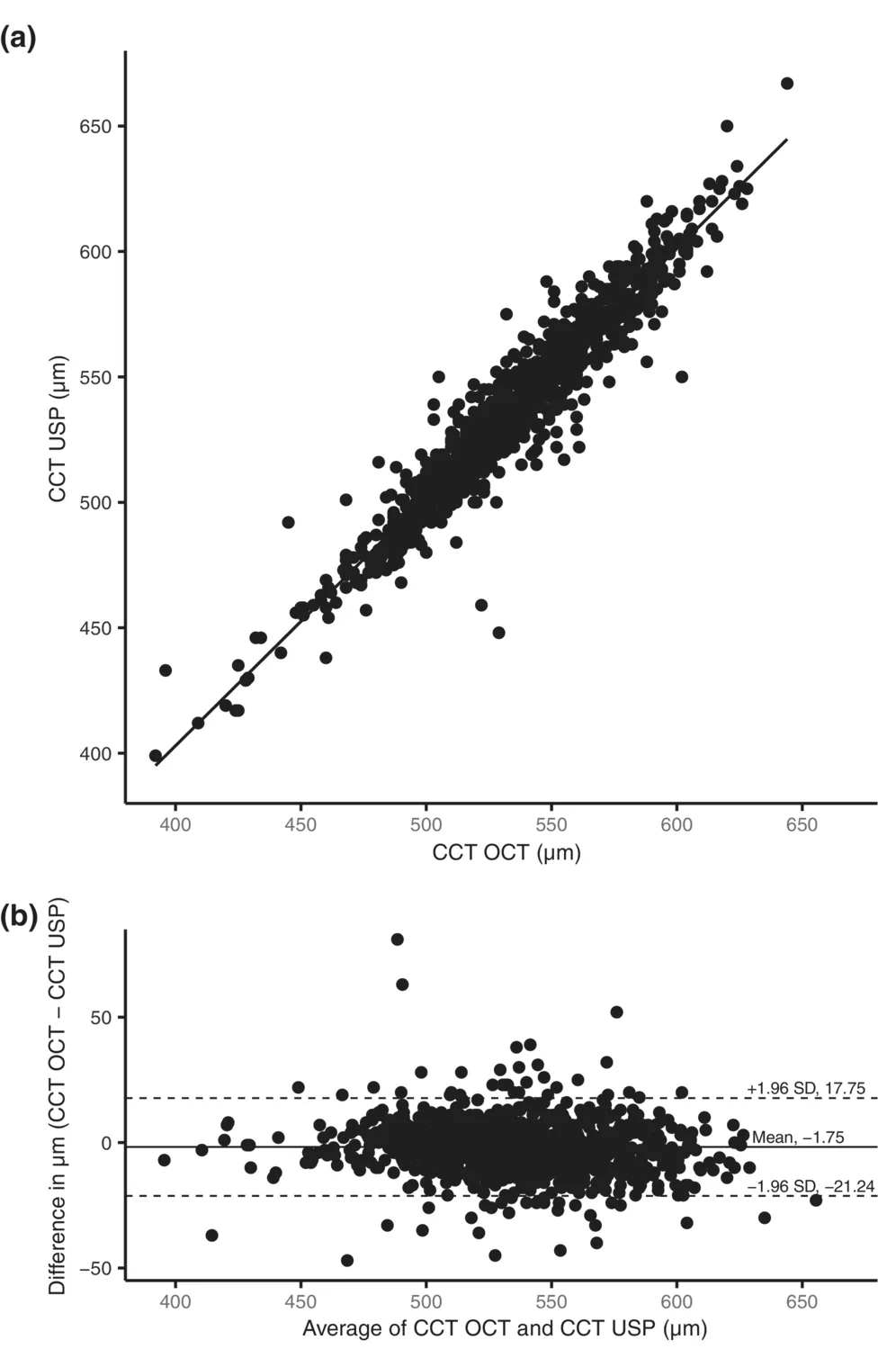
Scatter plot showing the distribution of CCT values using USP and OCT. The line of equality has been added for reference (a). Bland–Altman analysis between CCT with OCT and USP (b).

The CCT measured with OCT was correlated with IOP (*r* = 0.400 (iCare), *r* = 0.314 (GAT), *p* < 0.001), cup volume (*r* = −0.046, *p* = 0.014), rim area (*r* = 0.048, *p* = 0.011), RNFL (*r* = 0.041, *p* = 0.027) and refraction (*r* = 0.037, *p* = 0.047). In addition, CCT with USP was correlated with visual acuity (*r* = 0.064, *p* = 0.016). When examining the correlations between the CCT measurement differences and ophthalmic parameters, refractive error was correlated with the measurement difference (*r* = 0.138, *p* < 0.001). We also found that visual acuity showed a statistically significant correlation with the absolute measurement difference (*r* = 0.086, *p* = 0.003). The correlations with ophthalmic parameters are presented in Tables 2 and 3.

**TABLE 2:** Correlations between CCT and ophthalmic parameters.

	CCT OCT (μm)			CCT USP (μm)		
	*n*	*r*	*p*	*n*	*r*	*p*
Visual acuity/ETDRS	2840	−0.004	0.814	1403	0.064	**0.016**
Refraction/MSE (D)*	2846	0.037	**0.047**	1410	0.004	0.883
Disc area (HRT) (mm^2^)	2795	0.005	0.800	1375	0.016	0.557
Disc area (OCT) (mm^2^)	2814	0.028	0.141	1379	0.036	0.184
Cup volume (HRT) (mm^3^)*	2795	−0.049	**0.010**	1375	−0.041	0.125
Rim area (HRT) (mm^2^)	2795	0.048	**0.011**	1375	0.071	**0.008**
RNFL (OCT) (μm)	2837	0.041	**0.030**	1390	0.078	**0.004**
IOP (GAT) (mmHg)	1231	0.314	**< 0.001**	1260	0.322	**< 0.001**
IOP (iCare) (mmHg)	2846	0.400	**< 0.001**	1408	0.366	**< 0.001**

*Note*: The significant correlations are in bold.

Abbreviations: CCT, central corneal thickness; ETDRS, Early Treatment Diabetic Retinopathy Study; GAT, Goldman applanation tonometer; HRT, Heidelberg retina tomograph; MSE, mean spherical equivalent; OCT, optical coherence tomography; RNFL, retinal nerve fibre layer; USP, ultrasound pachymetry.

* Spearman's rho for non‐normally distributed variables.

**TABLE 3:** Correlations between the ophthalmic parameters and the differences and the absolute values of the differences in CCT measurements.

	CCT OCT − CCT USP			|CCT OCT − CCT USP|	
	*n*	*r*	*p*	*r*	*p*
Visual acuity/ETDRS	1234	−0.013	0.654	0.086	**0.003**
Refraction/mean spherical equivalent* (D)	1237	0.138	**< 0.001**	−0.023	0.411
Disc area (HRT) (mm^2^)	1205	−0.039	0.175	0.007	0.797
Disc area (OCT) (mm^2^)	1217	0.035	0.218	−0.017	0.542
Cup volume (HRT)* (mm^3^)	1205	0.031	0.285	0.002	0.948
Rim area (HRT) (mm^2^)	1205	−0.072	**0.013**	0.001	0.964
RNFL (OCT) (μm)	1227	0.068	**0.017**	−0.015	0.603
IOP (GAT) (mmHg)	1224	−0.056	**0.049**	0.000	0.987
IOP (iCare) (mmHg)	1234	−0.055	0.052	−0.043	0.134

*Note*: The significant correlations are in bold.

Abbreviations: CCT, central corneal thickness; ETDRS, Early Treatment Diabetic Retinopathy Study; GAT, Goldman applanation tonometer; HRT, Heidelberg retina tomograph; OCT, optical coherence tomography; RNFL, retinal nerve fibre layer; USP, ultrasound pachymetry.

* Spearman's rho for non‐normally distributed variables.

The linear regression slope (B) between IOP and CCT OCT was 0.041 mmHg/μm (95% CI 0.038–0.044, *p* < 0.001). In the multiple linear regression models, when adjusted for other ophthalmic parameters with significant correlation with CCT, the correlations between refraction and RNFL thickness with CCT OCT were no longer significant, even when the model was further adjusted for gender and glaucoma status (Table 4).

**TABLE 4:** Multiple regression analysis between CCT and ophthalmic parameters.

Independent variable	CCT OCT (μm) (*n* = 2763)				CCT USP (μm) (*n* = 1348)			
	*B*	*p*	95% CI		*B*	*p*	95% CI	
			Lower	Upper			Lower	Upper
*Model 1: Multiple regression adjusted for ophthalmic parameters with significant correlation with CCT*								
Visual acuity/ETDRS	1.164	0.853	−11.126	13.453	27.689	**0.006**	7.827	47.551
Refraction/MSE (D)	0.541	0.070	−0.044	1.126	0.279	0.563	−0.666	1.225
Cup volume (HRT) (mm^3^)	−13.497	**0.018**	−24.645	−2.349	−15.559	0.090	−33.576	2.458
Rim area (HRT) (mm^2^)	4.930	**0.015**	0.948	8.912	6.583	**0.045**	0.148	13.018
RNFL (OCT) (μm)	0.094	0.188	−0.046	0.233	0.316	**0.006**	0.091	0.542

Independent variable	CCT OCT (μm) (*n* = 2745)				CCT USP (μm) (*n* = 1341)			
	*B*	*p*	95% CI		*B*	*p*	95% CI	
			Lower	Upper			Lower	Upper
*Model 2: Multiple regression adjusted also for gender and glaucoma status*								
Visual acuity/ETDRS	5.937	0.348	−6.464	18.338	30.418	**0.003**	10.280	50.557
Refraction/MSE (D)	0.385	0.197	−0.201	0.971	0.194	0.689	−0.757	1.145
Cup volume (HRT) (mm^3^)	−14.731	**0.010**	−25.874	−3.587	−16.258	0.078	−34.355	1.838
Rim area (HRT) (mm^2^)	4.612	**0.023**	0.644	8.579	6.409	0.051	−0.034	12.852
RNFL (OCT) (μm)	0.134	0.061	−0.006	0.274	0.331	**0.004**	0.104	0.559

*Note*: The significant correlations are in bold.

Abbreviations: CCT, central corneal thickness; ETDRS, Early Treatment Diabetic Retinopathy Study; HRT, Heidelberg retina tomograph; MSE, mean spherical equivalent; OCT, optical coherence tomography; RNFL, retinal nerve fibre layer; USP, ultrasound pachymetry.

The CCT measured with OCT or USP showed no statistically significant correlation with glaucomatous damage. The mean CCT measured with OCT in subjects without glaucoma was 536 μm, in suspects 543 μm and in individuals with glaucoma 530 μm. There were no statistically significant differences between the groups (Figure 2).

**FIGURE 2:**
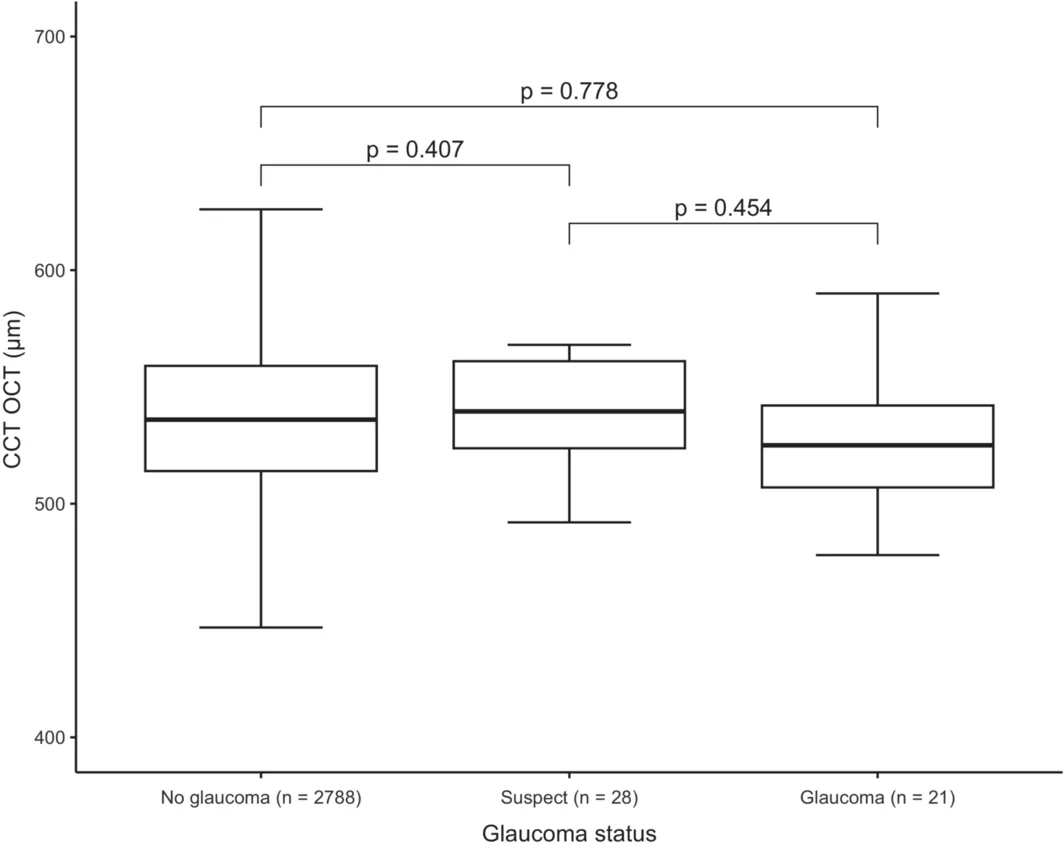
Boxplot image showing the mean and the distribution of CCT values for eyes with different glaucoma status and the statistical significance (*p*‐value) of the difference between the groups.

## 4 DISCUSSION

The mean CCT was almost identical between the methods, being 540.0 μm with OCT and 540.7 μm with USP. The CCT measured with OCT was highly correlated with IOP, but also positively correlated with rim area and RNFL and negatively with cup volume. However, no correlation was found between CCT and glaucoma.

### 4.1 Measurement methods

Limited and somewhat conflicting results have been presented in the literature regarding both the factors affecting as well as the agreement between the measurement methods of CCT. Ultrasonic pachymetry has been the method most used for measuring CCT and it has been referred to as the gold standard. A large meta‐analysis with over 100 studies reported CCT measurements using USP. The average CCT was 544 μm (Doughty & Zaman, 2000), which is close to our study. Gokcinar et al. performed a study where they measured CCT using six different devices, including OCT and USP. The 95% limits of agreement were the smallest between OCT and USP and the mean CCT values were 544.6 ± 29.6 μm for OCT and 545.3 ± 30.8 μm for USP (Gokcinar et al., 2019). Erdur et al. compared three methods for assessing CCT (Erdur et al., 2018). The mean CCT values were 555 ± 37 μm for USP, 554 ± 34 μm for noncontact specular microscopy (NSM) and 546 ± 34 μm for OCT and the correlation between USP and OCT was substantial (*r* = 0.975, *p* < 0.01). Ucak et al. performed CCT measurements using USP, noncontact tono/pachymetry, NSM, biometry, Scheimpflug‐based corneal topography and OCT (Ucak et al., 2021). They reported an overall strong correlation between the measurement methods. OCT and USP provided very similar results (552.6 ± 33.6 μm and 552.1 ± 35.7 μm). The Bland–Altman plot between OCT and USP demonstrated the lowest bias (0.53 μm). However, when Doğan and Ertan compared measurement methods for CCT, they found a mean CCT of 561 μm using USP and 543 μm with OCT (Doğan & Ertan, 2019). The 95% LoA were −42.9 to 8.0 μm and the mean difference was −17.4 μm (Doğan & Ertan, 2019). Our results in the Bland–Altman analysis showed a range of −21.2 μm to 17.8 μm, with a mean difference of −1.75 μm. However, the Pearson correlation was substantial (*r* = 0.927, *p* < 0.001), which is in agreement with the majority of the studies. Our research supports the idea that OCT and USP can be used interchangeably.

### 4.2 Correlations with ophthalmological parameters

In this present study, CCT was positively correlated with IOP. Several studies have shown similar results (Bikbov et al., 2023; Soleimanizad et al., 2017). Thicker CCT is associated with higher IOP readings. The association has been confirmed with both applanation and rebound tonometry. This is expected, as the two tonometry methods have been shown to be highly correlated in the NFBC population (Stoor et al., 2021). The associations between CCT and IOP can be described as reciprocal. That is, CCT affects the magnitude of IOP readings, whereas IOP may influence CCT through changes in the corneal endothelial pump function (Soleimanizad et al., 2017). When assessing the impact of CCT on IOP readings, a large meta‐analysis concluded that a 10% change in CCT would result in a 1.1 ± 0.6 mmHg change in IOP readings (Doughty & Zaman, 2000). Correspondingly, the Yard Eye Study concluded from several population‐based studies that an increase of 100 μm in CCT could add 1–3 mmHg to the IOP readings (Soleimanizad et al., 2017). However, a more substantial effect on IOP has also been reported. In a masked, prospective clinical trial, IOP was changed via cannulated anterior chamber before cataract surgery. The difference between measured and real IOP was significantly dependent on CCT. A 25 μm change in CCT from 550 μm illustrated an approximately 1 mmHg change in IOP (Kohlhaas et al., 2006). In the present study, a similar effect with a regression slope of 0.041 mmHg/μm (95% CI 0.038–0.044) between CCT OCT and IOP was observed.

When exploring the relationship between CCT and glaucoma, we discovered that CCT does not correlate with glaucoma in our study population. Our results are consistent with the Ural Eye and Medical Study (Bikbov et al., 2023). However, several studies have shown a correlation between CCT and glaucoma. The Ocular Hypertension Treatment Study (OHTS) investigated the correlation of CCT with glaucoma in patients with ocular hypertension (Gordon et al., 2002). Their findings indicated that a thinner CCT predicted the development of POAG. Another population‐based study found that there was a 41% increased risk of developing OAG per 40 μm thinner CCT (Leske et al., 2008). Los Angeles Latino Eye Study (LALES) found that patients with OAG had thinner CCT (Francis et al., 2008). Correspondingly, in Asian populations, glaucoma patients have been shown to have thinner CCT, whereas patients with OHT have thicker CCT (Aghaian et al., 2004).

There is ongoing debate on whether CCT is biologically related to glaucoma. The observed association may be mediated by the effect of CCT on IOP measurement. A simulation model mimicking the longitudinal population‐based datasets of LALES and OHTS has shown that thinner CCT is significantly associated with higher glaucoma incidence only when adjusted for measured IOP. Therefore, it is plausible that the observed CCT‐glaucoma associations are solely due to collider bias (Khawaja & Jansonius, 2022). Furthermore, a large genome‐wide association study for CCT found no evidence for a causal relationship between CCT and glaucoma in a Mendelian randomization experiment (Choquet et al., 2020). This would suggest that there is no true biological link between the two.

Despite the lack of correlation with glaucomatous damage in our study population, CCT did demonstrate a statistically significant positive correlation with rim area and RNFL and a negative correlation with cup volume. Although the p‐values are statistically significant, it is worth noting that the correlation coefficients are small and the associations may reflect anatomical differences between eyes and not necessarily impending ocular pathologies related to CCT. The Singapore Malay Eye Study found that in POAG patients, CCT was positively correlated with rim area. The study found no statistically significant correlation between CCT and disc size (Wu et al., 2011), and therefore the results are consistent with the present analysis. Correspondingly, Kaushik et al., (2006) found that CCT was positively correlated with RNFL and rim area, and negatively with cup area in ocular hypertension patients. Conflicting results have also been reported, since Cankaya et al. found that rim area and RNFL correlated negatively with CCT in healthy subjects, especially in women (Cankaya et al., 2008). Furthermore, correlations not found in our analysis have also been previously presented. For example, the Beijing Eye Study found a correlation between CCT and optic disc area (Zhang et al., 2008).

There is very limited knowledge on ophthalmic factors affecting inter‐method agreement on measuring CCT. The difference and the absolute difference in CCT with OCT and USP were calculated in the present study and the correlation of these differences with the ophthalmic parameters was analysed. The difference showed a statistically significant positive correlation with refraction. This indicates that CCT measured with OCT gives higher values compared with USP in more hyperopic patients. The absolute value was also used as the extent of the deviation between the methods was of particular interest. This correlates to the clinician's question: what are the properties of an eye in which good inter‐method agreement can be assumed. Visual acuity values with the ETDRS were found to correlate positively with CCT difference. Since the ETDRS values increase as visual acuity deteriorates, the inter‐method agreement between OCT and USP was further improved by better visual acuity. This correlation may be related to improved fixation stability during the OCT examination. The result further indicates that the measurements of OCT and USP may be used interchangeably, especially in patients with good visual acuity.

### 4.3 Strengths and limitations of the study

The Northern Finland Birth Cohort (NFBC1966) study has several strengths. It is a large population‐based birth cohort study which includes almost all children born in Northern Finland in 1966. It has managed to maintain a substantial number of active participants. The reasons for subject drop‐out from genetic epidemiological studies have been examined. Participants often find it inconvenient to attend in‐person sessions due to lack of time and transportation issues (Elskamp et al., 2012). Furthermore, the extended duration of birth cohort studies can represent a significant challenge, potentially leading to non‐participation. These reasons particularly affect a long‐term birth cohort study with prospective follow‐up. The participation rate of 60% in the NFBC Eye Study may be considered successful. Another strength is the meticulous randomization of the study population. Many studies primarily focus on investigating CCT in glaucoma patients or individuals with ocular hypertension. Our research significantly contributes data to studies conducted on a randomized, normal population, which is a well‐described homogenous population in terms of age, ethnicity and living environment.

The limitations of the NFBC Eye Study include the absence of pachymetry measurements in the remote research locations. The sample size for USP was only half of the OCT. However, with a sample size of 1413 for USP, it was still sufficient for demonstrating statistically significant results. Furthermore, since the study is population‐based, it includes only a few subjects with visual impairment. This may affect the evaluation of the correlation between visual acuity and inter‐method agreement. In addition, since the study was conducted as a cross‐sectional study, only limited conclusions on causative associations can be drawn.

In conclusion, our study highlights the interchangeability of CCT measurements with OCT and USP in a middle‐aged Caucasian population. The inter‐method agreement is further improved by good visual acuity. As expected, CCT had a substantial correlation with IOP but was also positively associated with ocular neural tissue parameters. However, we found no statistically significant correlation with glaucomatous damage.
